# Multimodality Imaging Diagnosis of Solitary Splenosis Including Shear-Wave Elastography in a Post-splenectomy Patient

**DOI:** 10.7759/cureus.108759

**Published:** 2026-05-12

**Authors:** Vignesh R, Venkata Sai, Abinesh Govindarajan, Darapaneni Chaitanya Kumar

**Affiliations:** 1 Radiology, Sri Ramachandra Institute of Higher Education and Research, Chennai, IND

**Keywords:** intra-abdominal mass, mri, radiological diagnosis, splenectomy, splenosis

## Abstract

Splenosis is an acquired condition characterized by heterotopic autotransplantation of splenic tissue following splenic trauma or splenectomy and may mimic intra-abdominal neoplastic lesions on imaging. We report the case of a 38-year-old female patient with a history of splenectomy who presented with right upper quadrant abdominal pain. Multimodality imaging revealed a solitary well-defined lesion in the splenic fossa demonstrating imaging characteristics comparable to native splenic tissue on ultrasonography, shear-wave elastography, and magnetic resonance imaging (MRI). Shear-wave elastography demonstrated homogeneous stiffness values, while MRI showed signal intensity and diffusion characteristics similar to those of splenic tissue, without aggressive imaging features. In the absence of histopathological or scintigraphic confirmation, the diagnosis strongly favored splenosis based on the characteristic imaging findings and clinical correlation. This case highlights the importance of recognizing the multimodality imaging appearance of splenosis to avoid unnecessary invasive diagnostic or surgical procedures.

## Introduction

Splenosis is a benign acquired condition characterized by heterotopic autotransplantation of viable splenic tissue following splenic trauma or splenectomy [1]. It occurs due to the dissemination and implantation of splenic fragments onto well-vascularized surfaces, most commonly within the peritoneal cavity [2]. Unlike accessory spleens, which are congenital, splenosis is an acquired phenomenon that may develop months to years after the initial insult and is frequently identified incidentally [3].

The true incidence of splenosis is likely underestimated; however, it is increasingly recognized because of the widespread use of advanced imaging modalities [4]. Most cases remain asymptomatic and are discovered incidentally during imaging performed for unrelated conditions [5]. Less commonly, patients may present with nonspecific abdominal symptoms, while rare complications such as obstruction or bleeding have also been reported [6].

Radiologically, splenosis may mimic neoplastic lesions, metastases, or lymphadenopathy, posing a significant diagnostic challenge [7]. Imaging modalities such as ultrasonography, computed tomography (CT), and magnetic resonance imaging (MRI) may suggest the diagnosis when lesions demonstrate imaging characteristics similar to those of normal splenic tissue [1,4]. Nuclear scintigraphy using technetium-99m-labeled heat-damaged red blood cells remains the most specific noninvasive diagnostic modality [7]. Accurate recognition is essential to avoid unnecessary invasive procedures such as biopsy or surgical excision [6].

In this report, we describe a case of solitary splenosis diagnosed using multimodality imaging, including shear-wave elastography and MRI, in a post-splenectomy patient presenting with right upper quadrant abdominal pain. This case highlights the role of noninvasive imaging in supporting the diagnosis and avoiding unnecessary intervention.

## Case presentation

A 38-year-old female patient presented with complaints of right upper quadrant abdominal pain for 15 days. The pain was acute in onset and was initially associated with a brief episode of vomiting lasting one day. There was no history of fever, jaundice, melena, weight loss, or loss of appetite.

Her past medical history was significant for a splenectomy performed in 2009 for multiple splenic cysts. She had no known history of diabetes mellitus, hypertension, tuberculosis, or malignancy. There was no history of alcohol consumption or smoking.

On clinical examination, the patient was hemodynamically stable. Abdominal examination revealed mild right upper quadrant tenderness without guarding or rigidity. No palpable mass was noted. Laboratory investigations, including complete blood count, liver function tests, and renal function tests, were within normal limits.

**Figure 1:**
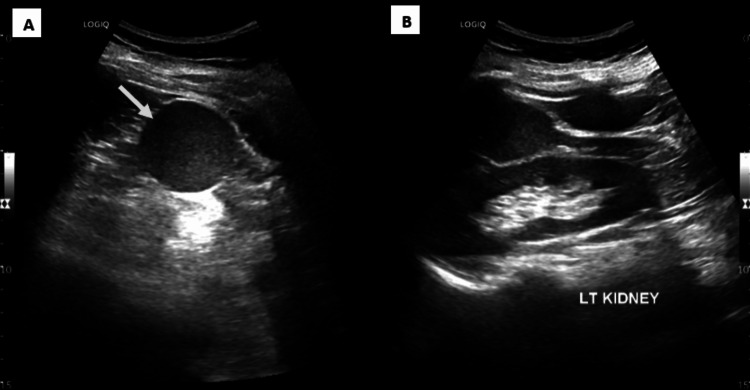
Ultrasonography of the abdomen (A) Absence of the native spleen in the splenic fossa, consistent with prior splenectomy, with a well-defined lesion (arrow) identified in the splenic fossa. (B) The same lesion (arrow) visualized adjacent to the upper pole of the left kidney, demonstrating homogeneous echogenicity comparable to splenic tissue, suggestive of solitary splenosis.

Ultrasonography of the abdomen was performed as the initial imaging modality. It revealed the absence of the spleen in the splenic fossa along with a well-defined lesion demonstrating echogenicity similar to splenic tissue (Figure 1).

**Figure 2:**
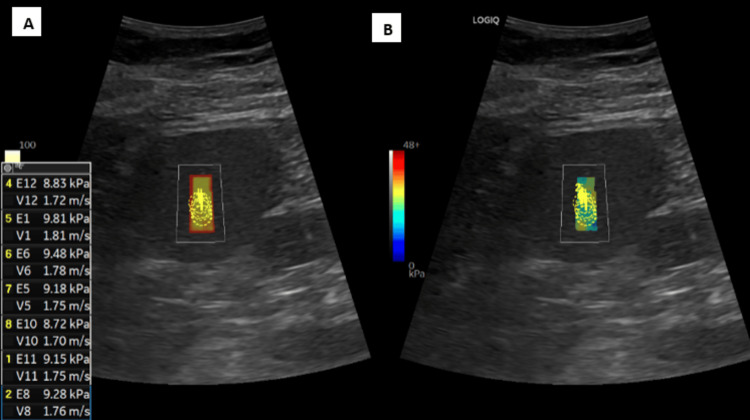
Ultrasound shear-wave elastography of the abdomen (A) B-mode grayscale image demonstrating a well-defined lesion in the splenic fossa. (B) Corresponding shear-wave elastography map demonstrating the same lesion with homogeneous stiffness distribution comparable to expected splenic tissue, without focal areas of increased stiffness or heterogeneity, supporting benign splenic tissue etiology and favoring splenosis.

Further evaluation with ultrasound elastography demonstrated homogeneous stiffness within the lesion, comparable to that of normal splenic tissue, without areas of increased stiffness or heterogeneity (Figure 2). 

**Figure 3:**
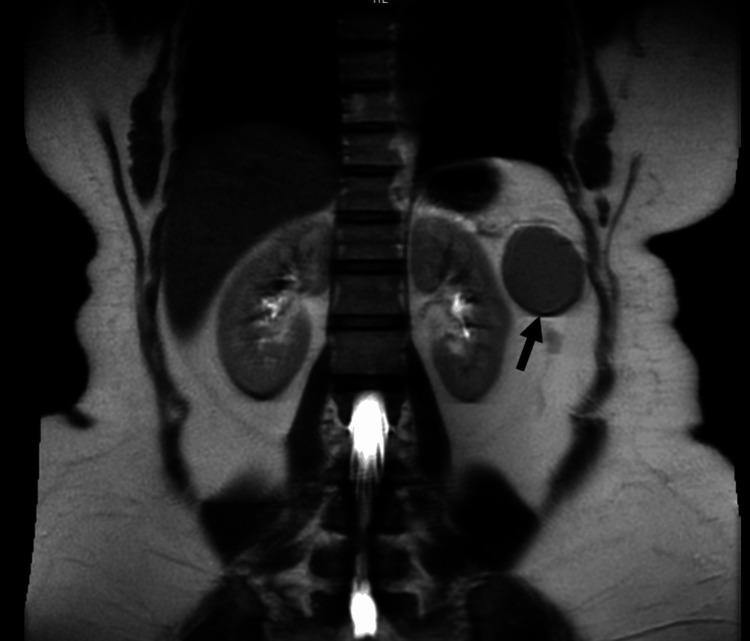
Abdominal MRI (coronal single-shot fast spin-echo (SSFSE) image) demonstrating a well-defined, homogeneous lesion (arrow) in the splenic fossa adjacent to the upper pole of the left kidney, showing signal intensity comparable to splenic tissue, suggestive of splenosis

**Figure 4:**
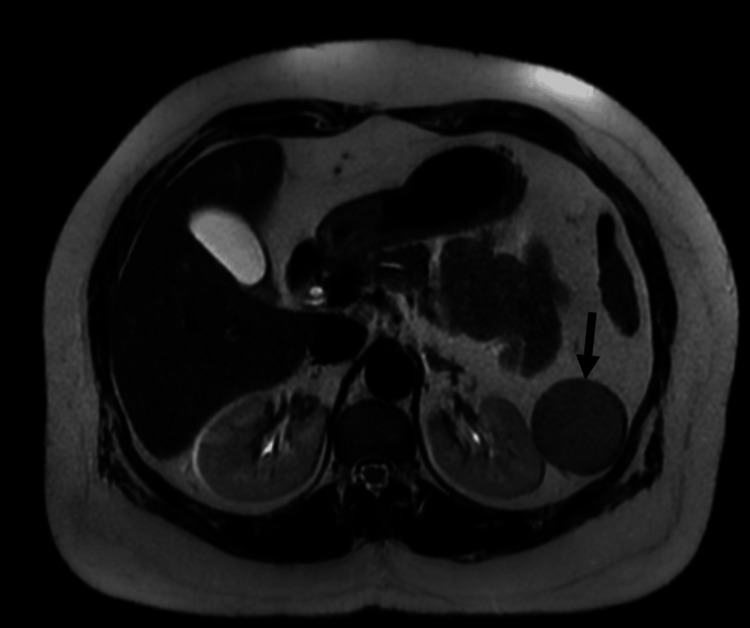
Abdominal MRI (axial single-shot fast spin-echo (SSFSE) image) demonstrating a well-circumscribed lesion (arrow) in the splenic fossa, showing homogeneous signal intensity similar to splenic tissue, without internal heterogeneity or aggressive features, favoring splenosis

**Figure 5:**
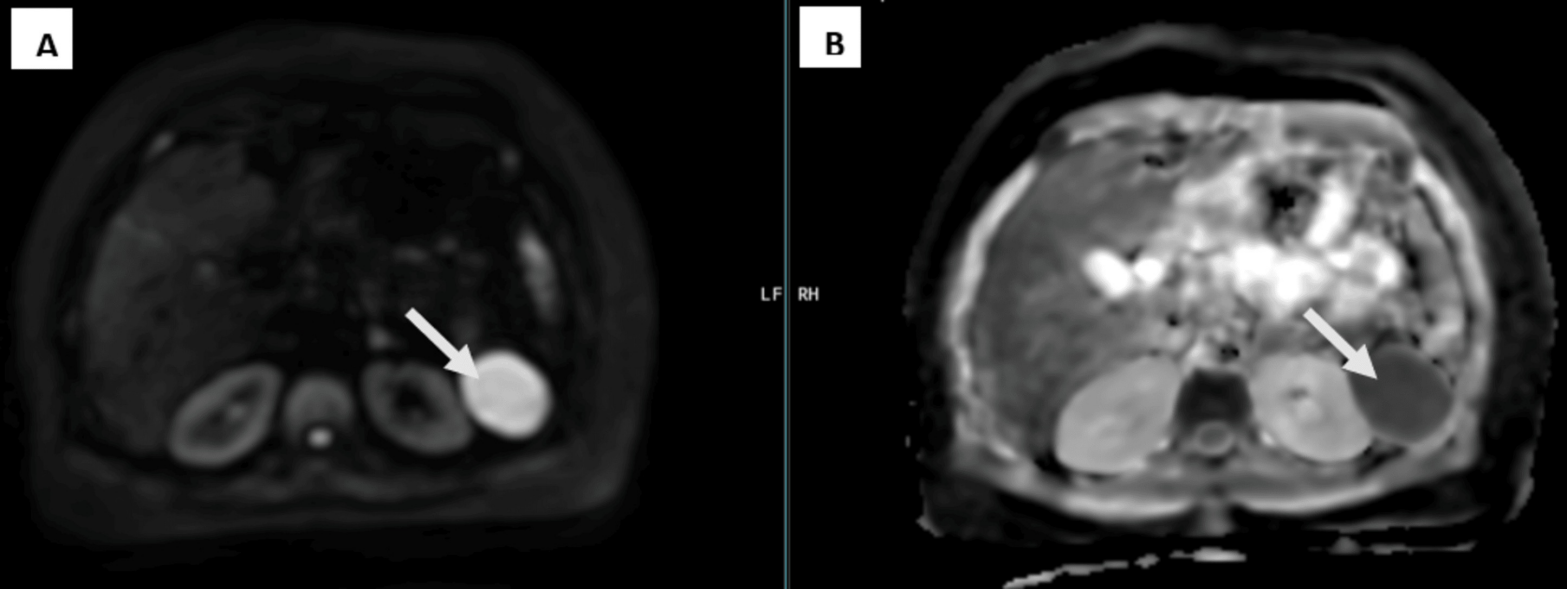
Abdominal MRI (diffusion-weighted imaging) (A) Diffusion-weighted image demonstrating a well-defined lesion (arrow) in the splenic fossa with high signal intensity. (B) Corresponding apparent diffusion coefficient (ADC) map showing low signal, with signal characteristics comparable to splenic tissue.

MRI of the abdomen was subsequently performed for further characterization. MRI demonstrated the absence of the native spleen and revealed a well-defined lesion measuring approximately 4.0 × 4.1 × 4.7 cm (AP × TR × CC) in the splenic fossa. The lesion demonstrated signal characteristics similar to native splenic tissue, appearing isointense on T1-weighted images and hyperintense on T2-weighted images. Diffusion-weighted imaging (DWI) showed high signal intensity with corresponding low apparent diffusion coefficient (ADC) values, without aggressive imaging features or evidence of pathological restricted diffusion (Figures 3-5).

Based on the patient’s history of prior splenectomy and the characteristic multimodality imaging findings, the diagnosis strongly favored splenosis. Nuclear scintigraphy was not performed because the radiological findings, in correlation with the clinical history, were considered sufficiently diagnostic.

## Discussion

Splenosis is a benign acquired condition resulting from autotransplantation of splenic tissue following splenic trauma or splenectomy. The underlying mechanism involves spillage of splenic pulp into adjacent cavities, where viable splenic fragments implant on serosal surfaces and derive their vascular supply from surrounding tissues [8]. These ectopic splenic implants retain functional characteristics and may persist for many years after the initial event, often remaining clinically silent.

The distribution of splenosis depends on the site of splenic injury. Common locations include the peritoneal cavity, omentum, mesentery, and splenic bed [9]. In the present case, the lesion was identified in the splenic fossa, consistent with the typical intra-abdominal distribution.

A key diagnostic challenge is the potential for splenosis to mimic malignant lesions, including peritoneal metastases, lymphadenopathy, gastrointestinal stromal tumors, organized collections, or accessory spleens [5]. Radiological evaluation is central to diagnosis. On CT and MRI, splenic nodules typically demonstrate imaging characteristics similar to normal splenic tissue, including a homogeneous appearance and comparable signal intensity patterns [10,11]. Accessory spleens are congenital, usually located near the splenic hilum, and derive their vascular supply from the splenic artery, whereas splenic implants acquire vascularity from surrounding tissues [10,11].

The role of elastography in splenosis has not been extensively described in the literature. In the present case, shear-wave elastography demonstrated homogeneous stiffness values ranging approximately from 8 to 10 kPa, comparable to expected splenic tissue. Although elastography is not specific for splenosis, it may serve as an adjunctive imaging tool supporting a benign splenic tissue etiology when interpreted in conjunction with conventional imaging findings and clinical history.

Nuclear scintigraphy using technetium-99m-labeled heat-damaged red blood cells is considered the most specific non-invasive modality for supporting the diagnosis of splenosis because of the selective uptake by functional splenic tissue [12]. In addition, awareness of atypical imaging presentations and diagnostic pitfalls is essential for accurate interpretation [13]. However, in cases with characteristic imaging findings and an appropriate clinical history, a confident non-invasive presumptive diagnosis may be established without additional testing.

Clinically, splenosis is usually asymptomatic and is most often detected incidentally during imaging performed for unrelated conditions [14]. When present, symptoms are typically nonspecific and depend on lesion size and location. In the present case, the splenic lesion was likely an incidental finding unrelated to the patient’s presenting symptoms. Similar incidental presentations have been widely reported [15].

From a management perspective, splenosis is a benign condition that typically requires no treatment unless symptomatic [9]. Surgical intervention is rarely indicated and is generally reserved for patients with complications such as bowel obstruction, infarction, hemorrhage, or significant mass effect [6]. Accurate recognition is essential to avoid unnecessary biopsy or surgical excision.

This case emphasizes the importance of considering splenosis in the differential diagnosis of intra-abdominal masses in patients with a prior splenectomy. Recognition of its characteristic imaging features allows a confident non-invasive presumptive diagnosis and helps prevent unnecessary invasive interventions.

## Conclusions

Splenosis is a benign and often incidental condition that should be considered in patients with a history of splenectomy who present with intra-abdominal lesions. Awareness of its characteristic imaging features, particularly its similarity to native splenic tissue on ultrasonography, shear-wave elastography, and MRI, can facilitate accurate diagnosis and prevent misinterpretation as malignancy. Correlation with clinical history is essential, and in appropriate clinical settings, non-invasive imaging findings may strongly support the diagnosis. Recognition of this entity helps avoid unnecessary invasive procedures or surgical interventions, as splenosis typically requires no treatment unless symptomatic.

## References

[1] Cheng B, Zhang X, Fu M, Gao P, Feng J, Wu F (2026). Case Report: Pelvic splenosis confused with malignancy-a reminder for differential diagnosis. Front Oncol.

[2] Möller K, Faiss S, Lim A, Jenssen C, Dietrich CF (2026). Splenoses and other ectopic and heterotopic splenic tissue: the use of long-lasting enhancement in contrast-enhanced ultrasound to avoid unnecessary intervention. Diagnostics (Basel).

[3] Younan G, Wills E, Hafner G (2015). Splenosis: a rare etiology for bowel obstruction-a case report and review of the literature. Case Rep Surg.

[4] Chapagain A, Yadav GK, Bhandari S, Devkota K, Adhikari B, Singh A, Bhattarai R (2024). Multiple intra-abdominal splenosis with imaging correlative findings: a case report and review of literature. Radiol Case Rep.

[5] Vernuccio F, Dimarco M, Porrello G, Cannella R, Cusmà S, Midiri M, Brancatelli G (2021). Abdominal splenosis and its differential diagnoses: what the radiologist needs to know. Curr Probl Diagn Radiol.

[6] Li X, Hu X, Wang P, Hu G, Zhou B, Cai J (2024). A large gastric splenosis mimicking gastrointestinal stromal tumor: a case report and literature review. Exp Ther Med.

[7] Ismail AM, Elbasateeny SS, Shafie A (2026). Laparoscopic management of abdominopelvic splenosis: a case report. Cureus.

[8] Fleming CR, Dickson ER, Harrison Jr EG (1976). Splenosis: autotransplantation of splenic tissue. American J Med.

[9] Fremont RD, Rice TW (2007). Splenosis: a review. South Med J.

[10] Lake ST, Johnson PT, Kawamoto S, Hruban RH, Fishman EK (2012). CT of splenosis: patterns and pitfalls. AJR Am J Roentgenol.

[11] Tsitouridis I, Michaelides M, Sotiriadis C, Arvaniti M (2010). CT and MRI of intraperitoneal splenosis. Diagn Interv Radiol.

[12] Wen Z, Edwards KW, States LJ, Zhuang H (2019). Heat-damaged red blood cell scintigraphy in helping interpretation of 68Ga-Dotatate PET/CT. Clin Nucl Med.

[13] Smoot T, Revels J, Soliman M (2022). Abdominal and pelvic splenosis: atypical findings, pitfalls, and mimics. Abdom Radiol (NY).

[14] Malik UF, Martin MR, Patel R, Mahmoud A (2010). Parenchymal thoracic splenosis: history and nuclear imaging without invasive procedures may provide diagnosis. J Clin Med Res.

[15] Wedemeyer J, Gratz KF, Soudah B (2005). Splenosis - an important differential diagnosis in splenectomized patients presenting with abdominal masses of unknown origin. Z Gastroenterol.

